# Automatic detection of break-over phase onset in horses using hoof-mounted inertial measurement unit sensors

**DOI:** 10.1371/journal.pone.0233649

**Published:** 2020-05-29

**Authors:** M. Tijssen, E. Hernlund, M. Rhodin, S. Bosch, J. P. Voskamp, M. Nielen, F. M. Serra Braganςa

**Affiliations:** 1 Department Population Health Sciences, Faculty of Veterinary Medicine, Utrecht University, Utrecht, The Netherlands; 2 Department of Anatomy, Physiology and Biochemistry, Swedish University of Agricultural Sciences, Uppsala, Sweden; 3 Inertia Technology B.V., Enschede, The Netherlands; 4 Department of Computer Science, Pervasive Systems Group, University of Twente, Enschede, The Netherlands; 5 Rosmark Consultancy, Wekerom, The Netherlands; 6 Department Clinical Sciences, Faculty of Veterinary Medicine, Utrecht University, Utrecht, The Netherlands; Massey University, NEW ZEALAND

## Abstract

A prolonged break-over phase might be an indication of a variety of musculoskeletal disorders and can be measured with optical motion capture (OMC) systems, inertial measurement units (IMUs) and force plates. The aim of this study was to present two algorithms for automatic detection of the break-over phase onset from the acceleration and angular velocity signals measured by hoof-mounted IMUs in walk and trot on a hard surface. The performance of these algorithms was evaluated by internal validation with an OMC system and a force plate separately. Seven Warmblood horses were equipped with two wireless IMUs which were attached to the lateral wall of the right front (RF) and hind (RH) hooves. Horses were walked and trotted over a force plate for internal validation while simultaneously the 3D position of three reflective markers, attached to lateral heel, lateral toe and lateral coronet of each hoof, were measured by six infrared cameras of an OMC system. The performance of the algorithms was evaluated by linear mixed model analysis. The acceleration algorithm was the most accurate with an accuracy between -9 and 23 ms and a precision around 24 ms (against OMC system), and an accuracy between -37 and 20 ms and a precision around 29 ms (against force plate), depending on gait and hoof. This algorithm seems promising for quantification of the break-over phase onset although the applicability for clinical purposes, such as lameness detection and evaluation of trimming and shoeing techniques, should be investigated more in-depth.

## Introduction

**Fig 1:**
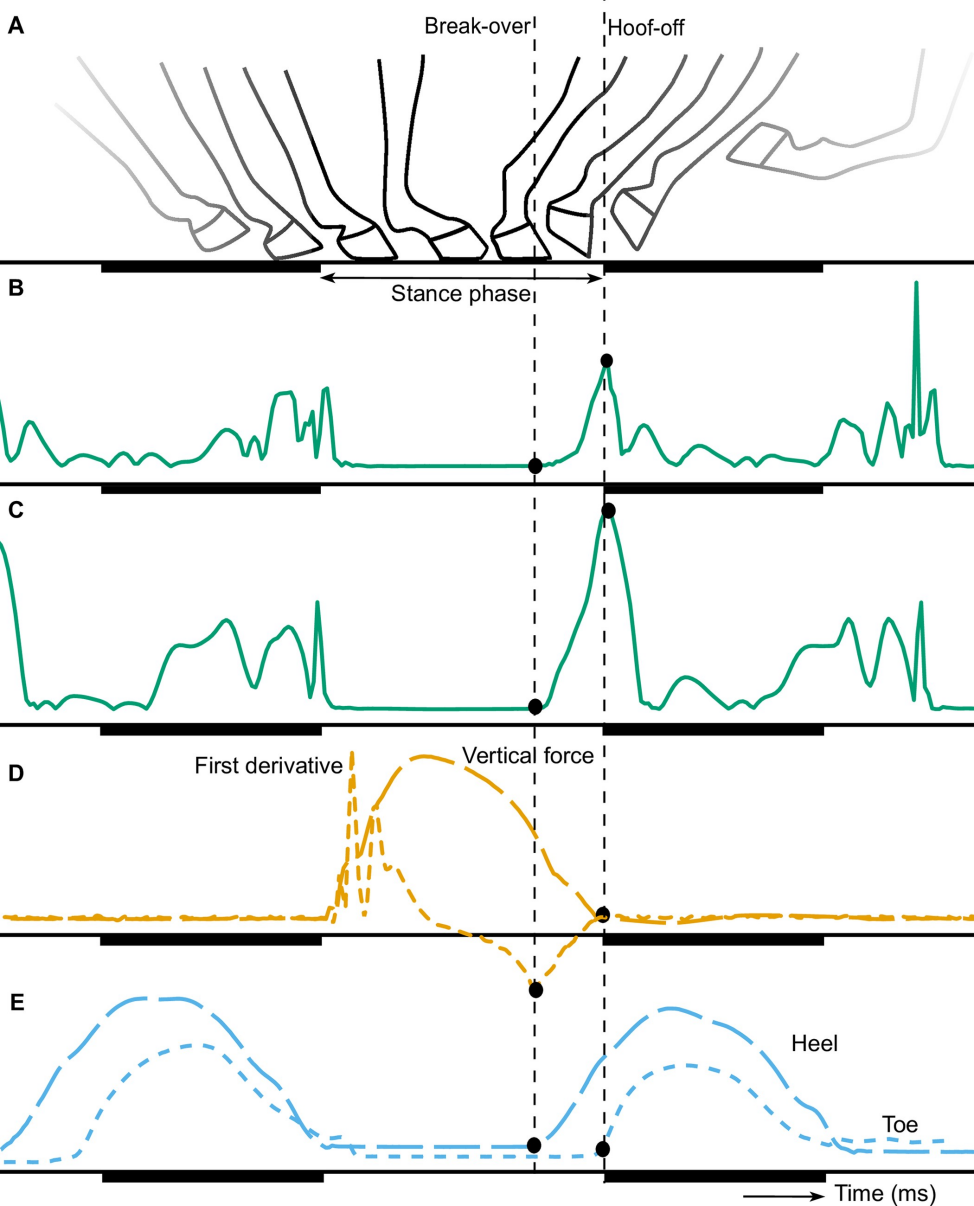
Generic illustration of the movement of the hoof (A), modified from Witte et al. [15], the signals of the acceleration (B), angular velocity (C), vertical force and first derivative of the vertical force (D), and vertical displacement of the heel and toe markers of the OMC system. The start of the break-over is depicted with the vertical dashed lines and the dots show the detected break-over from the different signals. The swing phase is underlined with a dark beam.

The break-over phase starts after the loading phase when the horse lifts its heel, causing a rotational movement around the toe, and ends with hoof-off [1, 2] as can be seen in Fig 1A. During this rotation, the body weight moves towards the toe; reducing the contact area with the ground and increasing the force on the toe and navicular bone. This increase in force results in high tensile forces on the muscles, ligaments and tendons [3, 4]. The ease of rotation of the hoof is affected by the toe length and hoof angle [4–6]. The break-over phase duration is the time between the start of the rotation and hoof-off. In general, this duration is around 20% of the stance duration in walk [7] but will be influenced by the gait and velocity of the horse, hoof shape and different surface properties. A prolonged break-over phase might increase the risk for development of navicular disease and tendon injury [3, 4, 8]. Prolongation can also be a result of a mechanical restriction or pain and thus an indication of an orthopedic disorder or lameness [4].

The break-over phase can be measured with kinematic methods such as optical motion capture (OMC) systems, and inertial measurement units (IMUs) since the rotation of the hoof can be derived from the output of these methods. The OMC systems measure the position of markers, placed on the hoof, over time and give information about the displacement of these markers. The IMUs can also be attached to the hoof and measure the acceleration and angular velocity in three directions over time [9, 10]. The displacement can be calculated by integration, although some noise in the input data and unknown initial conditions might affect the integration and lead to inaccurate results [11]. In a previous study by Clayton et al. (2000), the vertical force, measured by a force plate, was used to determine the loading rate on a limb [12]. The loading rate was defined as the slope of the vertical force right after hoof impact, in which the longitudinal force was decreasing. However, the slope of the decreasing vertical force, unloading rate, was not assessed in this study. In another study by Weishaupt et al. 2004, the typical shape of the vertical force curve was described in trot and a discrete kink after midstance was allocated to the breakover of the hoof [13].

In the current study, the three abovementioned techniques (IMU, OMC and force plate) are used to detect the start of the break-over phase. While OMC and force plate can be considered established methods, neither can be considered a perfect gold standard, as both measure different quantities (force versus position). The aim of this study was to present two algorithms for automatic detection of the break-over phase onset from the acceleration and angular velocity data measured by hoof-mounted IMUs in walk and trot on a hard surface. The performance of these algorithms was evaluated through internal validation with the OMC system and the vertical force separately.

## Materials and methods

### Data collection

The same data collection procedure was performed as described earlier [10, 14]. In short, measurements were performed with seven Warmblood horses (*Equus ferus caballus*; for further details see S1 Appendix).

These horses were equipped with two ProMove-mini wireless IMUs (Inertia-Technology B.V., Enschede, The Netherlands; for further details see S1 Appendix) which measured the low-*g* acceleration with a range of ±16 *g*, high-*g* acceleration with a range of ±400 *g*, angular velocity with a range of ±2000 º/s, and sampling frequency of 200 Hz. These IMUs were attached to the lateral wall of the right front (RF) and hind (RH) hooves with double sided and normal tape.

All horses were walked and trotted over a force plate (Z4852C, Kistler, Winterthur, Switzerland; for further details see S1 Appendix) to collect at least five valid force plate impacts for both front and hind hooves; each valid impact will be considered a trial in the further analysis.

Three reflective markers of the OMC system (Qualisys AB, Motion Capture System, Göteborg, Sweden; for further details see S1 Appendix) were attached to lateral heel, lateral toe and lateral coronet of each hoof with super glue. The 3D position of three reflective markers were measured with a sampling frequency of 200 Hz by six infrared cameras (ProReflex 240) of the OMC system (Qualisys AB, Motion Capture System, Göteborg, Sweden). The position of these markers and the acceleration and angular velocity signal measured by the IMU sensors was obtained simultaneously.

The systems were time synchronized as described earlier [10], for more details see S1 Appendix.

The original horse measurements were performed in compliance with the Dutch Act on Animal Experimentation and approved by the local ethics committee of Utrecht University. All horses were present for teaching purposes and these measurements were not considered additional animal experiments within the Dutch law at that time. Therefore, no specific experiment number is available.

### Data analysis

At the start of this study, data of the force plate, OMC system and IMUs were visually evaluated and a change in unloading rate of the vertical force signal was seen prior to hoof-off. To depict the unloading rate, the first derivative of the vertical force signal with respect to time was calculated and used during this study. The vertical force signal and the first derivative are depicted in Fig 1D.

#### OMC data

The collected OMC data were preprocessed by Inertia Technology B.V. and segmented in different trials corresponding with the force plate trials. The OMC data were analyzed and heel-off and toe-off time points, corresponding with the valid impact on the force plate, were selected by an algorithm described by Bragança et al. [14]. In short, the data from the toe and heel markers were filtered with a ‘maxflat filter’ with a cut-off frequency of 8 Hz. Then, the stance phase was detected by calculating the average variance of the signal using a moving window of 40 frames and allocating the moments with the lowest variance to the stance phase. Thereafter, the elevation of the markers was detected by performing a forward search to find the first frame where the marker was elevated by 1 mm, using the stance phase as a reference. This frame was allocated as the toe- and heel-off moments respectively. The same steps were performed with a backward search to find the toe- and heel-on moments. For this study, the break-over phase onset was determined as the heel-off time point.

#### Force plate data

The collected force plate data were preprocessed by Inertia Technology B.V.. The valid impacts were selected and cut into different trials; each trial consisted of at least one valid impact and sometimes two for consecutive impacts of the RF and RH hoof. The first derivative of the vertical force signal was calculated with a fourth order differentiator FIR filter with a passband frequency of 40 Hz and a stopband frequency of 100 Hz. The break-over phase onset was determined as the time point that the first derivative of the vertical force changed from decreasing to increasing values as can be seen in Fig 1D.

#### IMU data

The collected IMU data were preprocessed by Inertia Technology B.V. and cut into different trials corresponding with the force plate trials. The tri-axial acceleration and angular velocity signals were preprocessed by removing the offset drift and calculation of the Euclidean norm resulting in a one-directional acceleration and angular velocity signal. Thereafter, the swing phase was estimated to distinguish between consecutive steps by allocating the time points with a low variance as stance phase and the remaining time points as swing phase (for further details see companion paper [10]).

Next, we determined the break-over phase onset from the acceleration and angular velocity signal separately but by the same procedure. For both algorithms a threshold was developed to detect the start of the break-over phase. This threshold value was calculated from the signal mean (x) and signal standard deviation (s) of the stance phases. For every trial, this threshold value (T) was determined by:

T=x+1.96×s

The standard deviation was multiplied by 1.96 resulting in detection of the upper 2.5% of a normally distributed signal, to make sure that no random noise was detected.

The break-over phase onset was determined as the last time point that the signal was below the threshold value before hoof-off was detected.

### Performance evaluation

The time differences between the detection of the break-over phase onset of both algorithms were assessed with the OMC and force derivative separately as reference. The normality of the time differences was visually checked by examining the QQ plot and histogram in R (version 1.1.414, RStudio Inc, Boston, Massachusetts, USA). Thereafter, the distribution of the time differences was visualized to interpret the results and the performance of both algorithms was evaluated by a linear mixed model analysis.

For the linear mixed model analysis, the same model building and reduction procedure was performed as described previously [10]. In short, a linear mixed model analysis was performed with hoof, gait, number of trials and interaction term between hoof and gait as independent variables. A random intercept for every horse was included in the model. Model reduction was applied based on the AIC and residuals of each selected model were visually checked for any deviations of normality and homoscedasticity. The predicted value of the time difference between both algorithms with the OMC and force derivative separately were calculated for every combination of hoof and gait. The same procedure was performed for the time differences between the force derivative and the OMC system.

The performance of the algorithms was evaluated based on the predicted values and the width of the 95% confidence intervals of the time differences. The predicted value was deemed better if closer to zero which indicates a small difference between the algorithm and the reference measurement, i.e. a good accuracy. A positive predicted value indicates a delayed detection by the algorithm and a negative predicted value indicates a too early detection by the algorithm compared with the reference measurement. The width of the 95% confidence interval of the time difference was preferred to be small, which means that the time difference is measured precisely, i.e. a good precision. Schematic representations of these predicted values were used to visualize the accuracy and precision of the algorithms compared with the reference.

## Results

A total of 147 trials were analyzed: 75 trials of the right front (RF) hoof (36 in walk and 39 in trot) and 72 trials of the right hind (RH) hoof (34 in walk and 38 in trot). An overview of the analyzed trials is given elsewhere [10]. Preprocessed data of one measurement in trot can be seen in S1 Fig. The time differences between the detection of the break-over phase onset of both algorithms and the two reference methods (OMC and force plate) were normally distributed.

**Fig 2:**
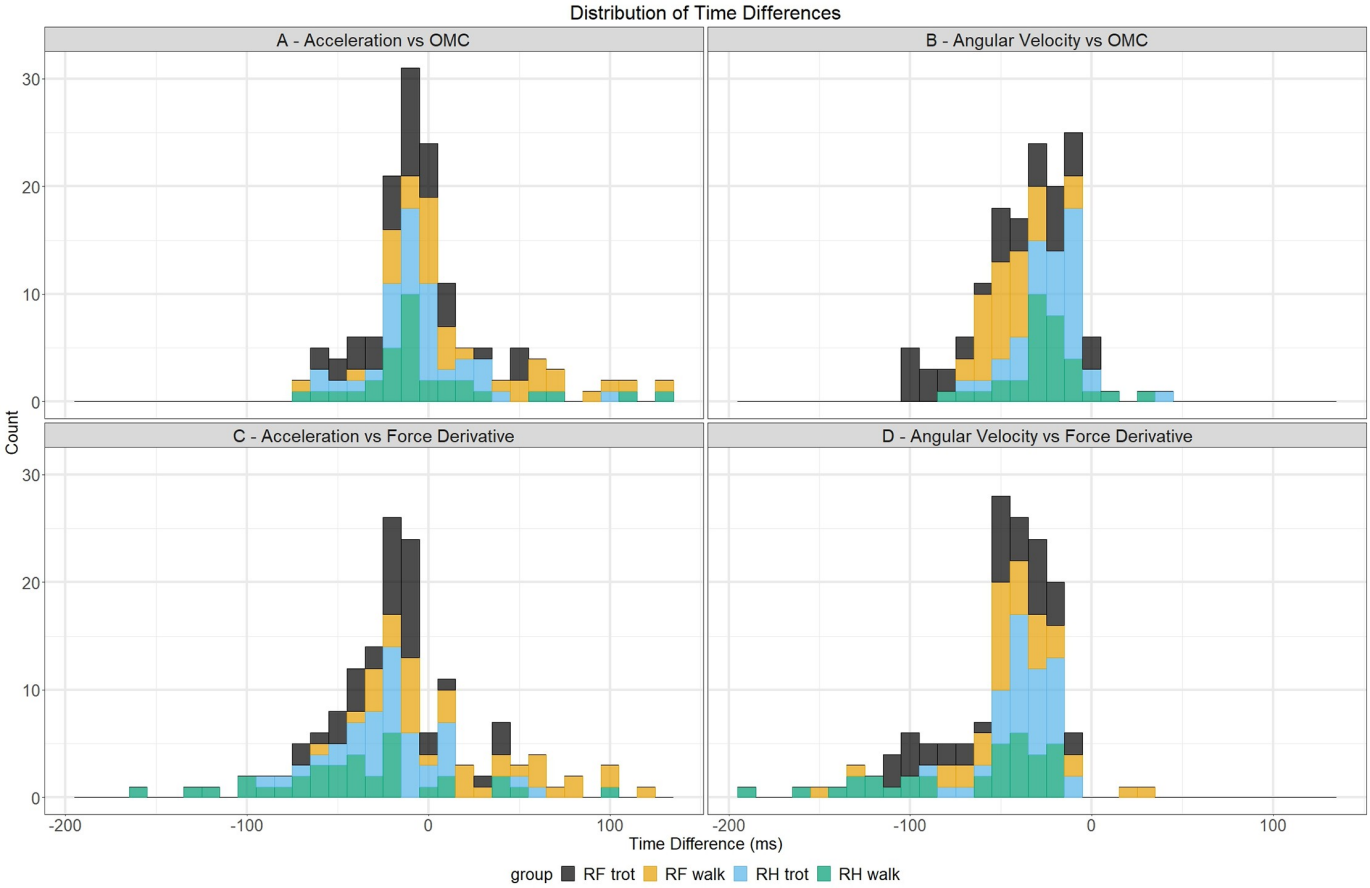
Distributions of time differences between both algorithms and reference methods for the break-over phase onset detection. Time differences with the OMC system are depicted in the upper row and time differences with the force derivative are depicted in the bottom row. The different hoof/gait combinations are depicted with their own color.

Time differences between both algorithms and the OMC system are depicted in the upper row of Fig 2. The distribution of the acceleration algorithm versus OMC system (Fig 2A) shows a bell shape curve ranging from -70 to 135 ms and a mean of 3.12 ms with higher values found for RH. The distribution of the angular velocity algorithm versus OMC system (Fig 2B) shows a smaller half bell shape curve, ranging from -100 to 10 ms, and a mean of -32.77 ms with lower values found for RF in trot. Time differences between both algorithms and the force derivative are depicted in the bottom row of Fig 2. The distribution of the acceleration algorithm versus force derivative (Fig 2C) shows a bell shape curve ranging from -155 to 125 ms and a mean of -12.18 ms with lower values found for RH in walk and higher values found for RF in walk. The distribution of the angular velocity algorithm versus force derivative (Fig 2D) shows a smaller right skewed curve ranging from -195 to 30 ms with a mean of -48.27 ms.

**Fig 3:**
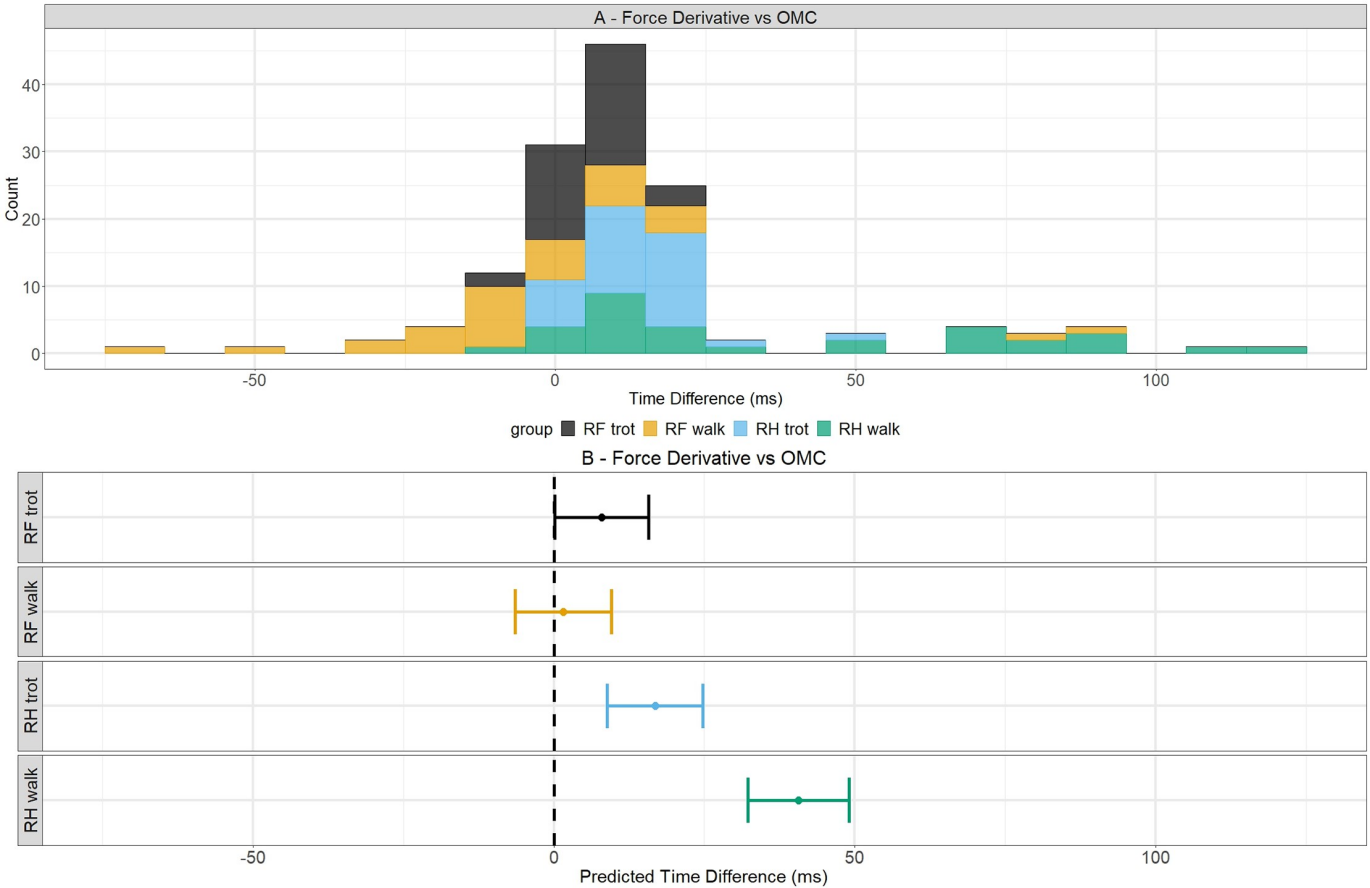
Time differences and predicted values of time differences between force derivative and OMC system for break-over phase onset detection. Time differences between the two reference methods are depicted in the upper figure with the different hoof/gait combinations depicted in their own color. In the bottom, the predicted values are indicated with dots for a certain hoof/gait combination and their 95% confidence intervals are shown by the whiskers. The dashed line indicates a predicted time difference of 0 ms.

Time differences between both reference methods are depicted in Fig 3A and show a bell shape curve, ranging from -75 to 120 ms and mean of 16.11 ms. Lower values were found for RH in walk and higher values for RF in walk.

### Linear mixed model analysis

The residuals of all selected linear mixed models were normally distributed and did not show homoskedasticity. Table 1 gives a summary of the results of the linear mixed model analysis.

**Table 1:** Linear mixed model results for the acceleration and angular velocity algorithm.

OMC system as reference					
			predicted value (ms)	lower CI (ms)	upper CI (ms)
Model 1: acceleration	walk	RF	22.57	10.43	34.72
		RH	2.03	-10.67	14.73
	trot	RF	-8.42	-20.08	3.23
		RH	-2.64	-14.61	9.34
Model 2: angular velocity	walk		-33.51	-47.08	-19.95
	trot		-31.56	-45.10	-18.02
		RF	-43.57	-57.11	-30.02
		RH	-21.50	-35.07	-7.94
Force derivative as reference					
			predicted value (ms)	lower CI (ms)	upper CI (ms)
Model 3: acceleration	walk	RF	20.25	5.76	34.74
		RH	-36.86	-51.65	-22.07
	trot	RF	-16.51	-30.65	-2.38
		RH	-16.73	-30.97	-2.50
Model 4: angular velocity	walk	RF	-43.39	-59.28	-27.50
		RH	-65.13	-81.15	-49.10
	trot	RF	-52.13	-67.87	-36.39
		RH	-34.48	-50.26	-18.69

The predicted values are determined in milliseconds (ms) and are deemed better if closer to zero. The upper and lower limits of the 95% confidence interval are determined in milliseconds (ms) and were preferred to be small.

### OMC as a reference

The models with the lowest AIC are presented in Table 1. The predicted values of the time difference between the acceleration algorithm and the OMC system (model 1) were best explained when hoof, gait and interaction term were included as fixed effect in the model with no random effect. The predicted values of the time difference between the angular velocity algorithm and the OMC system (model 2) were best explained when hoof and gait were included as fixed effect and horse as random effect in the model.

The results in Table 1 show that the predicted values of the time differences were closer to zero for the acceleration algorithm (model 1) compared with the angular velocity algorithm (model 2). For model 1, the predicted values were positive in walk and negative in trot indicating a delayed detection in walk and a too early detection in trot in contrast to model 2 for which all predicted values were negative indicating a too early detection. Also, the confidence intervals are smaller for the acceleration algorithm (model 1) compared with the angular velocity algorithm (model 2).

**Fig 4:**
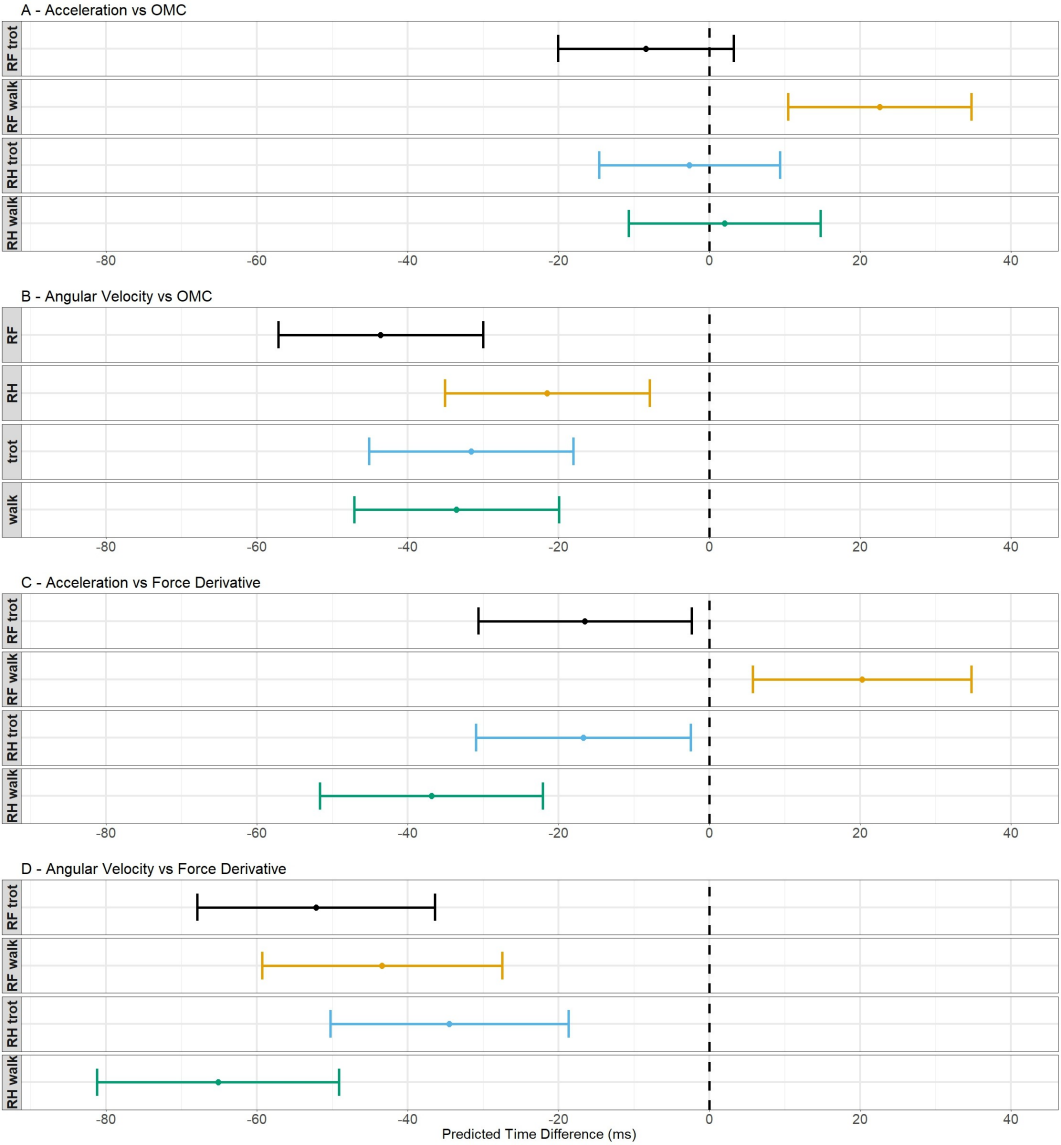
Schematic representation of the predicted values of the time differences and their 95% confidence intervals. The dots indicate the predicted value for a certain hoof/gait combination and the 95% confidence intervals are shown by the whiskers. The dashed line indicates a predicted time difference of 0 ms.

In Fig 4, the predicted values and their 95% confidence intervals are shown for all models. For model 1 (Fig 4A), these values are shown for every hoof/gait combination because this model needs an interaction term to explain the data. The predicted values and their 95% confidence intervals of model 2 (Fig 4B) are shown for walk versus trot and the RF hoof versus the RH hoof because this model did not need an interaction term to explain the data. For model 1, the predicted values for RH are located closer to zero compared with the RF. All confidence intervals contain both positive and negative values except for the interval of the RF in walk. For model 2, the predictive values of the gaits are located closer to each other than the values of both hooves. The predictive value of RH is located closest to zero and the value of RF is located most distant from zero.

These results indicate that the agreement with the OMC was better for the acceleration algorithm with an accuracy of between -8.42 and 22.57 ms depending on the gait and hoof and a precision around 24.24 ms.

### Force derivative as a reference

The predicted values of the time differences between the acceleration algorithm and the force plate (model 3) and the angular velocity algorithm and the force plate (model 4) were best explained when in the model hoof, gait and interaction term were included as fixed effect and horse as random effect.

The results in Table 1 show that the predicted values were smaller for the acceleration algorithm (model 3) compared with the angular velocity algorithm (model 4). The predictive values of both models were all negative, indicating a too early detection, except for RF in walk of model 3. Also, the widths of the 95% confidence intervals were smaller for the acceleration algorithm (model 3).

For model 3 (Fig 4C) and model 4 (Fig 4D), these values are shown for every hoof/gait combination because this model needs an interaction term to explain the data. For model 3, the predictive values and confidence intervals are negative except for the RF in walk which has a positive predictive values and completely positive confidence interval. The values of both hooves are located closer to zero in trot compared to walk. For model 4, the predictive values and all confidence intervals are completely negative. The predictive value of RH in trot is located the closest to zero and the value of RH in walk is located the most distant from zero.

These results indicate that the agreement with the force derivative was better for the acceleration algorithm, which agrees with the results found for the validation with the OMC system. The accuracy of this algorithm was found between -36.86 and 20.25 ms, depending on gait and hoof, and the precision was around 28.83 ms.

### Force derivative versus OMC system

The predicted values of the time differences between the two references methods, force derivative and OMC system, were best explained when the model contained a fixed effect for hoof and gait, an interaction term and no random horse effect.

The results in Table 2 show that all predicted values are positive, indicating a delayed detection with the force derivative compared to the OMC system. The value for RH in walk was the most located from zero. The confidence intervals were all completely positive, except for the interval of the RF in walk.

**Table 2:** Linear mixed model results for the reference methods, OMC system and force derivative.

Force derivative vs OMC system				
		predicted values (ms)	lower Cl (ms)	upper Cl (ms)
walk	RF	1.57	9.56	-6.44
	RH	40.62	49.01	32.24
trot	RF	7.97	15.77	0.18
	RH	16.81	24.71	8.90

The predicted values are determined in milliseconds (ms) and are deemed better if closer to zero. The lower and upper limits of the 95% confidence interval are determined in milliseconds (ms) and were preferred to be small.

In Fig 3B, the predicted values and their 95% confidence intervals are shown for every hoof/gait combination because this model needs an interaction term to explain the data. For the RF hoof in both gaits, the predicted values were closer to zero in contrast to the RH in walk for which the predicted value was the most distant from zero. All the confidence intervals were completely positive except for the interval of the RF in walk.

## Discussion

Two algorithms are described to automatically detect the break-over phase onset from the acceleration and angular velocity signals measured with hoof-mounted IMUs in walk and trot on a hard surface. Results of the internal validation show that the acceleration algorithm was the most accurate with an accuracy between -9 and 23 ms and a precision around 24 ms with assessment against the OMC system and an accuracy between -37 and 20 ms and a precision around 29 ms with assessment against the force plate, depending on gait and hoof.

Both models needed a hoof, gait and interaction term to explain the predicted values of the time differences. The hoof effect might be explained by the fact that the shape of the hind- and front hooves are different; the hind hoof angle is steeper and the hoof is narrower, which results in a different hoof-unrollment pattern compared with the front hoof [6, 16]. The hoof-unrollment pattern might be affected by the velocity of the horse which explained the gait effect. The interaction term might be explained by the fact that hoof-unrollment patterns of hind- and front hooves might change differently over different gaits.

This is the first study that reports the use of the slope of the decreasing vertical force, unloading rate, for detection of the break-over phase onset. When the two established methods, OMC system and force plate, were assessed with each other, the predicted values of the time difference showed a time difference of 7.97 and 16.81 ms for respectively RF and RH in trot and 1.57 and 40.62 ms for respectively RF and RH in walk. This shows that the break-over phase onset detection based on the first derivative of the vertical force signal did not agree closely to the OMC system for all hoof/gait combinations. Based on the results of the current study, it’s not possible to conclude which method detects the break-over phase onset most accurately. However, the OMC system might be better suited for detecting of rotational movement around the toe while the force plate might be better suited for detection of sudden hoof contact moments [10]. Although, to determine toe-off and heel-off timings with the OMC system, an arbitrary threshold is needed in contrast to the force derivative.

During this study, no clear effect of stance duration on the performance of the break-over phase onset detection was seen. Stance durations can be found in S1 Table of the companion paper [10]. With the force derivative as reference, the angular velocity algorithm did not show a better performance in walk or trot, although the acceleration algorithm did show a better performance in trot compared to walk. With the OMC system as reference, the angular velocity algorithm did show a better performance in trot compared to walk, although the acceleration algorithm did not show a better performance in walk or trot.

The results of this study show that the acceleration algorithm was the most accurate algorithm to detect the break-over phase onset which was not as expected since the hoof was not yet lifted from the ground and no big acceleration change occurred. This might be a result of calculation of the Euclidean norm; an increasing angular velocity in one direction might become concealed by a decrease in angular velocity in another direction. For clinical applications, such as evaluation of hoof trimming, testing different shoeing techniques [6, 17–19] and lameness detection [20], it might be beneficial to investigate the acceleration and angular velocity in three directions separately since rotation direction and maximal angle of rotation change with different hoof shapes and when horses are lame.

Besides measuring the rotation direction, the break-over duration might be a helpful addition for these applications. An increased break-over duration was found at mild lameness, even before lameness could be detected with the naked eye and before the stance duration of the lame limb increased [21]. In the current study, break-over durations were not evaluated by statistical analysis because calculation of these durations depends on the break-over onset as well as hoof-off detection per measurement method. Both detections are performed with their corresponding accuracy and precision, where the accuracy and precision of hoof-off detections is discussed in the companion paper [10]. An overview of the break-over durations within each measurement method is given in S1–S4 Tables with their relative duration as percentage of the corresponding stance duration.

Break-over onset timing relative to the stance phase might also be of importance. In a previous study by Clayton et al. (2000), coffin joint moment and force plate data showed that the center of pressure began to move forward in a relatively early stage of the stance phase, initiating an early break-over phase onset in the lame limb [12].Further research should be performed to investigate the possible wider applicability of these algorithms, for instance on different surfaces. On a hard surface, the hoof remains flat on the ground until heel-off, in contrast to a soft surface on which the toe rotates into the surface before heel-off [22]. This will probably make break-over phase onset detection more difficult with these algorithms. Furthermore, reliability of these algorithms should be revalued in different situations, such as sound and lame conditions or before and after hoof trimming.

## Conclusion

Two algorithms were presented to automatically detect the start of the break-over phase from the acceleration and angular velocity data measured with hoof-mounted IMUs in walk and trot on a hard surface. Internal validations against the OMC system and unloading rate, measured by the force derivative, were performed separately. The acceleration algorithm appeared to perform best with an accuracy between -9 and 23 ms and a precision around 24 ms (with the OMC system as reference), and an accuracy between -37 and 20 ms and a precision around 29 ms (with the force plate as reference), depending on gait and hoof. These algorithms seem promising for the onset of the break-over phase quantification. However, a more extensive validation process should be performed with more data and additional horses. Furthermore, the applicability of these algorithms for clinical purposes, such as lameness detection and evaluation of trimming and shoeing techniques, should be investigated more in-depth, including on different surfaces.

## Supporting information

S1 Appendix Additional information. Document with additional information about the population, data collection and synchronization of all the measurement systems.
(DOCX)

S2 Appendix Dataset. Excel file with hoof-on, hoof-off and break-over data.
(XLSX)

S1 Fig Preprocessed signals of the force plate, vertical force (A) and the first derivative of the vertical force (B), the acceleration (C) and angular velocity (D) signals of the IMU, and vertical displacement signals of the heel and toe markers of the OMC system (E) from one hoof from one measurement in trot. The hoof-on events are depicted with upward-pointing triangle markers, hoof-off events are depicted with downward-pointing triangle markers and break-over onset events are depicted with diamond shaped markers. For the OMC data, break-over onset events are depicted were the heel of the hoof leaves the ground and hoof-off events are depicted were the toe of the hoof leaves the ground.
(TIF)

S1 Table Break-over durations per trial in milliseconds (ms) and relative to stance duration (%) for right front hoof in walk. Tables with break-over durations. Tables with break-over durations per trial in milliseconds (ms) and relative to corresponding stance duration (%) as detected with the acceleration and angular velocity algorithms, force derivative and OMC system for every hoof and gait combination.
(DOCX)

S2 Table Break-over durations per trial in milliseconds (ms) and relative to stance duration (%) for right hind hoof in walk. Tables with break-over durations. Tables with break-over durations per trial in milliseconds (ms) and relative to corresponding stance duration (%) as detected with the acceleration and angular velocity algorithms, force derivative and OMC system for every hoof and gait combination.
(DOCX)

S3 Table Break-over durations per trial in milliseconds (ms) and relative to stance duration (%) for right front hoof in trot. Tables with break-over durations. Tables with break-over durations per trial in milliseconds (ms) and relative to corresponding stance duration (%) as detected with the acceleration and angular velocity algorithms, force derivative and OMC system for every hoof and gait combination.
(DOCX)

S4 Table Break-over durations per trial in milliseconds (ms) and relative to stance duration (%) for right hind hoof in trot. Tables with break-over durations. Tables with break-over durations per trial in milliseconds (ms) and relative to corresponding stance duration (%) as detected with the acceleration and angular velocity algorithms, force derivative and OMC system for every hoof and gait combination.
(DOCX)

## References

[1] Thomason JJ, Peterson ML. Biomechanical and mechanical investigations of the hoof-track interface in racing horses. Vet Clin North Am Equine Pract. 2008;24(1):53–77. 18314036 10.1016/j.cveq.2007.11.007

[2] Starke SD, Clayton HM. A universal approach to determine footfall timings from kinematics of a single foot marker in hoofed animals. PeerJ. 2015;3:e783. 26157641 10.7717/peerj.783 PMC4493675

[3] Clayton HM. Comparison of the stride of trotting horses trimmmed with a normal and a broken-back hoof axis. Proceedings 7th American Association of Equine Practitioners1987 p. 289–98.

[4] Clayton HM, Sigafoos R, Curle RD. Effect of three shoe types on the duration of breakover in sound trotting horses. Journal of Equine Veterinary Science. 1990;11(2):129–32.

[5] Wilson A, Agass R, Vaux S, Sherlock E, Day P, Pfau T, et al Foot placement of the equine forelimb: Relationship between foot conformation, foot placement and movement asymmetry. Equine Vet J. 2016;48(1):90–6. 25523459 10.1111/evj.12378

[6] Keegan KG, Satterley JM, Skubic M, Yonezawa Y, Cooley JM, Wilson DA, et al Use of gyroscopic sensors for objective evaluation of trimming and shoeing to alter time between heel and toe lift-off at end of the stance phase in horses walking and trotting on a treadmill. American Journal of Veterinary Research. 2005;66(12):2046–54. 16379645 10.2460/ajvr.2005.66.2046

[7] Chateau H, Degueurce C, Denoix JM. Three-dimensional kinematics of the equine distal forelimb: effects of a sharp turn at the walk. Equine Vet J. 2005;37(1):12–8. 15651728 10.2746/0425164054406946

[8] Glade MJ, Salzman RA. Effects of toe angle on hoof growth and contraction in the horse. Journal of Equine Veterinary Science. 1985;5(1):45–50.

[9] Bosch S, Serra Braganca F, Marin-Perianu M, Marin-Perianu R, van der Zwaag BJ, Voskamp J, et al EquiMoves: A Wireless Networked Inertial Measurement System for Objective Examination of Horse Gait. Sensors (Basel). 2018;18(3). 10.3390/s18030850 PMC5877382 29534022

[10] Tijssen M. A method for automatic hoof-event detection in horses based on hoof-mounted inertial measurement units. Submitted to PLOS ONE. 2020a, companion paper. 10.1371/journal.pone.0233266 PMC7269263 32492034

[11] Pfau T, Witte TH, Wilson AM. A method for deriving displacement data during cyclical movement using an inertial sensor. J Exp Biol. 2005;208(Pt 13):2503–14. 15961737 10.1242/jeb.01658

[12] Clayton HM, Schamhardt HC, Willemen MA, Lanovaz JL, Colborne GR. Kinematics and ground reaction forces in horses with superficial digital flexor tendinitis. American Journal of Veterinary Research. 2000;61(2):191–6. 10685692 10.2460/ajvr.2000.61.191

[13] Weishaupt MA, Wiestner T, Hogg HP, Jordan P, Auer JA. Vertical ground reaction force-time histories of sound Warmblood horses trotting on a treadmill. Vet J. 2004;168(3):304–11. 15501148 10.1016/j.tvjl.2003.08.007

[14] Braganca FM, Bosch S, Voskamp JP, Marin-Perianu M, Van der Zwaag BJ, Vernooij JCM, et al Validation of distal limb mounted inertial measurement unit sensors for stride detection in Warmblood horses at walk and trot. Equine Vet J. 2017;49(4):545–51. 27862238 10.1111/evj.12651 PMC5484301

[15] Witte TH, Knill K, Wilson AM. Determination of peak vertical ground reaction force from duty factor in the horse (Equus caballus). J Exp Biol. 2004;207(Pt 21):3639–48. 15371472 10.1242/jeb.01182

[16] Van Heel MCV, Moleman M, Barneveld A, Van Weeren PR, Back W. Changes in location of centre of pressure and hoof-unrollment pattern in relation to an 8-week shoeing interval in the horse. Equine Vet J. 2005;37(6):536–40. 16295931 10.2746/042516405775314925

[17] Page BT, Hagen TL. Breakover of the hoof and its effect on stuctures and forces within the foot. Journal of Equine Veterinary Science. 2002;22(6):258–64.

[18] Spaak B, van Heel MC, Back W. Toe modifications in hind feet shoes optimise hoof-unrollment in sound Warmblood horses at trot. Equine Vet J. 2013;45(4):485–9. 23094947 10.1111/j.2042-3306.2012.00659.x

[19] Van Heel MCV, Van Weeren PR, Back W. Shoeing sound Warmblood horses with a rolled toe optimises hoof-unrollment and lowers peak loading during breakover. Equine Vet J. 2006;38(3):258–62. 16706282 10.2746/042516406776866471

[20] Moorman VJ, Reiser RF 2nd, Mahaffey CA, Peterson ML, McIlwraith CW, Kawcak CE. Use of an inertial measurement unit to assess the effect of forelimb lameness on three-dimensional hoof orientation in horses at a walk and trot. Am J Vet Res. 2014;75(9):800–8. 25157883 10.2460/ajvr.75.9.800

[21] Moorman VJ, Reiser RF 2nd, Peterson ML, McIlwraith CW, Kawcak CE. Effect of forelimb lameness on hoof kinematics of horses at a walk. Am J Vet Res. 2013;74(9):1192–7. 23977891 10.2460/ajvr.74.9.1192

[22] Hernlund E, Egenvall A, Roepstorff L. Kinematic characteristics of hoof landing in jumping horses at elite level. Equine Vet J Suppl. 2010(38):462–7. 21059046 10.1111/j.2042-3306.2010.00187.x

