# Supplementary material for: Automatic detection of break-over phase onset in horses using hoof-mounted inertial measurement unit sensors
Source: PLoS One. 2020 May 29;15(5):e0233649. doi: 10.1371/journal.pone.0233649 (PMC7259550; doi:10.1371/journal.pone.0233649)
Supplement: S1 Appendix — Document with additional information about the population, data collection and synchronization of all the measurement systems. (DOCX) [file pone.0233649.s001.docx]

# **Additional information**

## **Horses**

The measurements were performed for a previous study performed by Braganςa et al. 2017 (1). Measurements were performed with seven Warmblood horses, six mares and one gelding, with a body mass ranging from 506 to 608 kg (mean 564.4 kg), age ranging from five to twenty-one years (mean 7.5 years) and height at withers ranging from 1.58 to 1.75 m (mean 1.65 m).

## **Data collection**

The walking speed of the horses was measured with two pairs of photoelectric sensors placed before and after the force plate at a distance of two meters.

### **Optical motion capture setup**

Three markers were used for the motion measurements; these markers were spherical passive markers with a diameter of 12.5 mm. The 3D position of the three markers was measured with six infrared cameras (ProReflex 240) of the optical motion capture (OMC) system (Qualisys AB, Motion Capture System, Göteborg, Sweden). The cameras were placed around the force plate in such way that the vertical displacement of the markers was visible. Movements of the markers was recorded with a sampling frequency of 200 Hz and with a relative precision of 1.9 mm after calibration (2). The collected OMC data was used in another study for break-over detection (3) but was needed for time synchronization in this paper.

### **Force plate setup and synchronization**

The force plate (Z4852C, Kistler, Winterthur, Switzerland) was covered with a five mm rubber mat. The analogue force plate signal was fed to an A/D converter with a sampling frequency of 1000 Hz and connected to the Qualisys Track Manager (QTM) software (Qualisys AB, Motion Capture System, Göteborg, Sweden) of the OMC system. The QTM software down sampled the force plate signal with a frequency of 200 Hz and removed the response time lag to obtain time synchronization with the OMC system ((1); for further details see page 134 of the QTM manual (4)).

### **Inertial measurement unit setup and synchronization**

The ProMove-mini wireless inertial measurement units (IMUs) (Inertia-Technology B.V., Enschede, The Netherlands) weighted 20 g and were set to a sampling frequency of 200 Hz. The sensors measured the tri-axial acceleration, angular velocity (gyroscope) and magnetic field intensity (compass) over time with a precision of 100 ns (2). The collected data was stored on the onboard 2 Gb microSD card during measurements and was retrieved after each trial.

Time synchronization between the IMUs and OMC system is described by Bosch et al. (2). In short, two reflective markers were attached above and below the IMU attached to the cannon bone of the horse (data is used for another study (1)). The correlation coefficient was calculated between the position data of these reflective markers and the angular velocity signal measured with this IMU. The time shift between the OMC system and the IMU was indicated by a maximum in the correlation coefficient. To improve this calculation, interpolation was used which resulted in a time synchronization estimated better than 500 µs (2)).

# **References**

1. Braganca FM, Bosch S, Voskamp JP, Marin-Perianu M, Van der Zwaag BJ, Vernooij JCM, et al. Validation of distal limb mounted inertial measurement unit sensors for stride detection in Warmblood horses at walk and trot. Equine Vet J. 2017;49(4):545-51.

2. Bosch S, Serra Braganca F, Marin-Perianu M, Marin-Perianu R, van der Zwaag BJ, Voskamp J, et al. EquiMoves: A Wireless Networked Inertial Measurement System for Objective Examination of Horse Gait. Sensors (Basel). 2018;18(3).

3. Tijssen M. Break-over detection using hoof-mounted inertial measurements units. Submitted to Journal of Experimental Biology. 2020b, companion paper.

4. Qualisys AB. Qualisys Track Manager - User Manual. Gothenborg, Sweden2011.
