# Supplementary figures and images for: Automatic detection of break-over phase onset in horses using hoof-mounted inertial measurement unit sensors
Source: PLoS One. 2020 May 29;15(5):e0233649. doi: 10.1371/journal.pone.0233649 (PMC7259550; doi:10.1371/journal.pone.0233649)

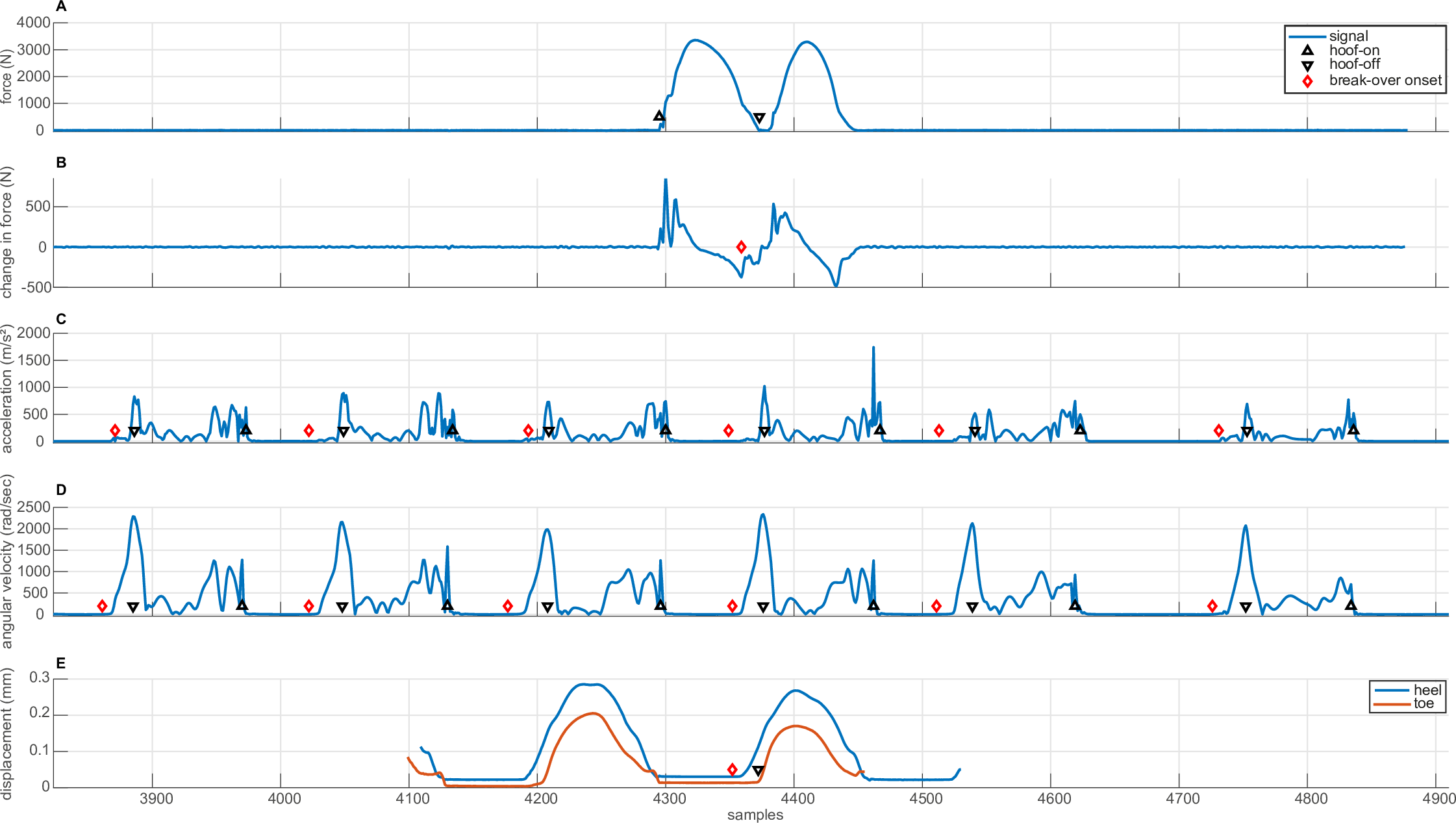

Supplement: S1 Fig — Preprocessed signals of the force plate, vertical force (A) and the first derivative of the vertical force (B), the acceleration (C) and angular velocity (D) signals of the IMU, and vertical displacement signals of the heel and toe markers of the OMC system (E) from one hoof from one measurement in trot. The hoof-on events are depicted with upward-pointing triangle markers, hoof-off events are depicted with downward-pointing triangle markers and break-over onset events are depicted with diamond shaped markers. For the OMC data, break-over onset events are depicted were the heel of the hoof leaves the ground and hoof-off events are depicted were the toe of the hoof leaves the ground. (TIF) [file pone.0233649.s003.tif]
