# Supplementary material for: Automatic detection of break-over phase onset in horses using hoof-mounted inertial measurement unit sensors
Source: PLoS One. 2020 May 29;15(5):e0233649. doi: 10.1371/journal.pone.0233649 (PMC7259550; doi:10.1371/journal.pone.0233649)
Supplement: S1 Table — Tables with break-over durations. Tables with break-over durations per trial in milliseconds (ms) and relative to corresponding stance duration (%) as detected with the acceleration and angular velocity algorithms, force derivative and OMC system for every hoof and gait combination. (DOCX) [file pone.0233649.s004.docx]

**Table S1: Break-over durations per trial in milliseconds (ms) and relative to stance duration (%) for right front hoof in walk**

| Break-over duration in ms (%) for right front hoof in walk | | | | | | | | | |
| --- | --- | --- | --- | --- | --- | --- | --- | --- | --- |
| horse ID | trial | Acceleration | | Angular Velocity | | Force Derivative | | OMC | |
| 1 | 1 | 140 | (18.92) | 170 | (22.97) | 145 | (19.33) | 90 | (12.59) |
|  | 2 | 150 | (21.13) | 170 | (23.78) | 140 | (19.18) | 60 | (8.82) |
|  | 3 | 150 | (20.00) | 175 | (23.33) | 165 | (21.71) | 60 | (8.76) |
|  | 4 | 110 | (14.57) | 210 | (27.63) | 160 | (20.78) | 80 | (11.68) |
|  | 5 | 155 | (19.75) | 195 | (25.16) | 150 | (19.23) | 70 | (9.59) |
| 2 | 1 | 140 | (17.50) | 175 | (21.74) | 130 | (16.15) | 110 | (13.84) |
|  | 2 | 90 | (11.11) | 170 | (20.99) | 125 | (15.53) | 100 | (12.74) |
|  | 3 | 25 | (3.05) | 185 | (22.02) | 135 | (16.56) | 135 | (17.09) |
|  | 4 | 115 | (13.69) | 185 | (21.76) | 135 | (16.07) | 120 | (14.20) |
|  | 5 | 215 | (24.43) | 175 | (19.89) | 150 | (17.14) | 50 | (6.17) |
| 3 | 1 | 80 | (10.13) | 170 | (21.79) | 125 | (15.82) | 65 | (8.72) |
|  | 2 | 70 | (8.97) | 175 | (22.15) | 50 | (6.29) | 100 | (12.99) |
|  | 3 | 140 | (19.18) | 160 | (21.62) | 115 | (15.65) | 105 | (14.29) |
|  | 4 | 105 | (14.09) | 160 | (20.78) | 120 | (15.29) | 125 | (15.82) |
|  | 5 | 55 | (7.75) | 175 | (22.88) | 135 | (17.53) | 100 | (13.89) |
| 4 | 1 | 175 | (23.18) | 225 | (29.80) | 170 | (21.66) | 45 | (6.98) |
|  | 2 | 165 | (19.88) | 230 | (27.38) | 170 | (20.00) | 30 | (4.35) |
|  | 3 | 190 | (23.60) | 200 | (24.24) | 170 | (20.00) | 20 | (2.94) |
|  | 5 | 155 | (18.90) | 240 | (27.75) | 170 | (19.54) | 35 | (4.73) |
|  | 6 | 155 | (19.38) | 240 | (28.40) | 175 | (20.59) | 90 | (11.69) |
| 5 | 1 | 45 | (5.81) | 185 | (24.18) | 135 | (17.42) | 35 | (5.43) |
|  | 2 | 185 | (22.16) | 200 | (23.95) | 165 | (19.53) | 55 | (7.80) |
|  | 3 | 45 | (5.70) | 185 | (23.42) | 150 | (18.99) | 100 | (14.18) |
|  | 4 | 145 | (16.67) | 190 | (21.84) | 160 | (18.39) | 15 | (2.10) |
|  | 5 | 95 | (11.24) | 205 | (24.12) | 55 | (6.43) | 30 | (4.23) |
| 6 | 1 | 140 | (17.61) | 180 | (21.95) | 115 | (14.38) | 45 | (6.29) |
|  | 4 | 135 | (17.31) | 175 | (22.88) | 100 | (12.90) | 95 | (12.58) |
|  | 5 | 145 | (17.47) | 185 | (22.70) | 105 | (12.65) | 55 | (7.24) |
|  | 7 | 45 | (5.39) | 170 | (19.88) | 115 | (13.94) | 105 | (12.96) |
|  | 8 | 100 | (14.29) | 155 | (20.81) | 180 | (24.66) | 35 | (5.43) |
|  | 9 | 135 | (17.88) | 175 | (23.49) | 210 | (27.63) | 60 | (8.70) |
| 7 | 1 | 95 | (13.01) | 100 | (13.79) | 85 | (11.49) | 30 | (4.41) |
|  | 2 | 110 | (15.38) | 105 | (15.00) | 90 | (12.59) | 0 | - |
|  | 3 | 30 | (3.92) | 105 | (13.91) | 90 | (11.61) | 30 | (4.38) |
|  | 4 | 35 | (4.93) | 105 | (15.00) | 95 | (13.19) | 35 | (5.56) |
|  | 5 | 115 | (16.79) | 120 | (17.78) | 80 | (10.88) | 25 | (3.91) |

The break-over duration is determined as the time between break-over phase onset and hoof-off for the force plate, acceleration and angular velocity algorithms. For the OMC system, the break-over duration is determined as the time between heel-off and toe-off. The stance duration is determined as the time between hoof-on and hoof-off for the force plate, acceleration and angular velocity algorithms. For the OMC system, the stance duration is determined as the time between heel-on and toe-off. Break-over duration as percentage of the corresponding stance duration is given between brackets.
