# Supplementary material for: Automatic detection of break-over phase onset in horses using hoof-mounted inertial measurement unit sensors
Source: PLoS One. 2020 May 29;15(5):e0233649. doi: 10.1371/journal.pone.0233649 (PMC7259550; doi:10.1371/journal.pone.0233649)
Supplement: S2 Table — Tables with break-over durations. Tables with break-over durations per trial in milliseconds (ms) and relative to corresponding stance duration (%) as detected with the acceleration and angular velocity algorithms, force derivative and OMC system for every hoof and gait combination. (DOCX) [file pone.0233649.s005.docx]

**Table S2: Break-over durations per trial in milliseconds (ms) and relative to stance duration (%) for right hind hoof in walk**

| Break-over duration in ms (%) for right hind hoof in walk | | | | | | | | | |
| --- | --- | --- | --- | --- | --- | --- | --- | --- | --- |
| horse ID | trial | Acceleration | | Angular Velocity | | Force Derivative | | OMC | |
| 1 | 1 | 150 | (19.74) | 165 | (21.85) | 55 | (7.14) | 60 | (8.76) |
|  | 2 | 145 | (19.59) | 160 | (21.19) | 105 | (13.64) | 60 | (8.82) |
|  | 3 | 165 | (21.85) | 190 | (24.84) | 80 | (10.26) | 50 | (7.30) |
|  | 4 | 90 | (12.00) | 125 | (16.56) | 85 | (10.97) | 65 | (9.29) |
|  | 5 | 175 | (23.18) | 135 | (17.65) | 130 | (16.46) | 65 | (9.29) |
| 2 | 1 | 130 | (16.99) | 190 | (25.00) | 60 | (8.00) | 25 | (3.70) |
|  | 2 | 160 | (21.05) | 165 | (21.57) | 70 | (8.75) | 145 | (18.01) |
|  | 3 | 135 | (17.20) | 185 | (23.57) | 60 | (7.45) | 40 | (5.67) |
|  | 4 | 120 | (15.19) | 210 | (25.93) | 60 | (7.32) | 55 | (7.48) |
|  | 5 | 225 | (25.86) | 240 | (28.40) | 55 | (6.08) | 125 | (15.15) |
| 3 | 1 | 125 | (16.67) | 155 | (20.53) | 120 | (16.00) | 45 | (6.52) |
|  | 2 | 140 | (18.54) | 160 | (21.05) | 110 | (14.67) | 45 | (6.57) |
|  | 3 | 125 | (17.48) | 125 | (17.24) | 115 | (15.75) | 40 | (6.02) |
|  | 4 | 130 | (17.69) | 150 | (20.41) | 115 | (15.65) | 40 | (5.88) |
|  | 5 | 130 | (18.31) | 140 | (19.86) | 100 | (13.70) | 35 | (5.30) |
| 4 | 1 | 145 | (18.71) | 140 | (17.95) | 125 | (15.82) | 65 | (8.78) |
|  | 2 | 200 | (25.00) | 170 | (21.25) | 120 | (15.00) | 55 | (7.43) |
|  | 5 | 55 | (6.67) | 190 | (22.49) | 135 | (15.88) | 55 | (7.19) |
|  | 6 | 200 | (25.32) | 170 | (20.99) | 135 | (16.46) | 55 | (7.24) |
| 5 | 1 | 100 | (12.66) | 165 | (20.75) | 120 | (15.09) | 0 | - |
|  | 2 | 150 | (18.07) | 170 | (20.24) | 130 | (15.20) | 45 | (6.04) |
|  | 3 | 175 | (21.60) | 185 | (22.70) | 140 | (16.87) | 65 | (8.78) |
|  | 4 | 170 | (20.12) | 170 | (19.88) | 125 | (14.20) | 0 | - |
|  | 5 | 25 | (2.91) | 175 | (20.47) | 65 | (7.30) | 45 | (5.84) |
| 6 | 1 | 155 | (20.26) | 195 | (24.84) | 170 | (21.79) | 130 | (17.11) |
|  | 5 | 160 | (18.71) | 175 | (20.11) | 100 | (11.24) | 115 | (14.02) |
|  | 7 | 215 | (22.51) | 205 | (22.16) | 130 | (13.76) | 0 | - |
|  | 8 | 185 | (24.50) | 195 | (25.49) | 65 | (8.44) | 160 | (21.19) |
|  | 9 | 165 | (22.15) | 165 | (21.43) | 60 | (7.64) | 160 | (20.38) |
| 7 | 1 | 50 | (6.71) | 160 | (20.92) | 105 | (13.46) | 65 | (9.15) |
|  | 2 | 130 | (17.69) | 140 | (19.18) | 100 | (13.42) | 40 | (5.97) |
|  | 3 | 75 | (10.07) | 135 | (17.76) | 105 | (13.46) | 30 | (4.35) |
|  | 4 | 160 | (22.07) | 130 | (18.06) | 90 | (12.24) | 40 | (5.97) |
|  | 5 | 95 | (13.57) | 130 | (17.57) | 85 | (10.97) | 35 | (5.11) |

The break-over duration is determined as the time between break-over phase onset and hoof-off for the force plate, acceleration and angular velocity algorithms. For the OMC system, the break-over duration is determined as the time between heel-off and toe-off. The stance duration is determined as the time between hoof-on and hoof-off for the force plate, acceleration and angular velocity algorithms. For the OMC system, the stance duration is determined as the time between heel-on and toe-off. Break-over duration as percentage of the corresponding stance duration is given between brackets.
