# Supplementary material for: Automatic detection of break-over phase onset in horses using hoof-mounted inertial measurement unit sensors
Source: PLoS One. 2020 May 29;15(5):e0233649. doi: 10.1371/journal.pone.0233649 (PMC7259550; doi:10.1371/journal.pone.0233649)
Supplement: S3 Table — Tables with break-over durations. Tables with break-over durations per trial in milliseconds (ms) and relative to corresponding stance duration (%) as detected with the acceleration and angular velocity algorithms, force derivative and OMC system for every hoof and gait combination. (DOCX) [file pone.0233649.s006.docx]

**Table S3: Break-over durations per trial in milliseconds (ms) and relative to stance duration (%) for right front hoof in trot**

| Break-over duration in ms (%) for right front hoof in trot | | | | | | | | | |
| --- | --- | --- | --- | --- | --- | --- | --- | --- | --- |
| horse ID | trial | Acceleration | | Angular Velocity | | Force Derivative | | OMC | |
| 1 | 1 | 140 | (36.36) | 120 | (30.00) | 70 | (17.95) | 60 | (16.22) |
|  | 3 | 95 | (27.54) | 120 | (33.33) | 70 | (19.44) | 45 | (14.52) |
|  | 4 | 125 | (34.72) | 125 | (33.33) | 70 | (20.00) | 30 | (9.09) |
|  | 6 | 150 | (42.86) | 125 | (35.21) | 70 | (20.00) | 25 | (9.26) |
|  | 7 | 90 | (25.71) | 125 | (34.25) | 60 | (17.14) | 50 | (14.49) |
| 2 | 1 | 35 | (12.96) | 180 | (57.14) | 70 | (22.22) | 20 | (7.69) |
|  | 4 | 90 | (26.47) | 195 | (52.70) | 65 | (20.63) | 20 | (7.27) |
|  | 5 | 50 | (15.87) | 200 | (56.34) | 75 | (21.74) | 30 | (10.34) |
|  | 6 | 55 | (19.64) | 180 | (48.65) | 65 | (20.31) | 20 | (7.27) |
|  | 7 | 110 | (32.35) | 195 | (52.70) | 70 | (21.21) | 25 | (9.43) |
| 3 | 1 | 75 | (22.39) | 80 | (24.24) | 55 | (16.92) | 20 | (6.90) |
|  | 2 | 110 | (32.84) | 80 | (23.19) | 75 | (21.74) | 65 | (19.12) |
|  | 3 | 100 | (27.40) | 95 | (22.35) | 70 | (19.72) | 60 | (17.39) |
|  | 4 | 110 | (31.88) | 100 | (29.85) | 65 | (19.40) | 40 | (12.70) |
|  | 5 | 75 | (23.08) | 80 | (23.88) | 60 | (18.46) | 60 | (18.46) |
|  | 7 | 45 | (14.75) | 80 | (23.88) | 65 | (19.40) | 60 | (19.35) |
| 4 | 1 | 75 | (22.73) | 185 | (50.00) | 75 | (20.83) | 45 | (15.25) |
|  | 2 | 105 | (31.82) | 120 | (39.34) | 75 | (21.74) | 10 | (4.17) |
|  | 3 | 105 | (30.43) | 130 | (41.27) | 70 | (20.00) | 0 | - |
|  | 4 | 115 | (31.94) | 175 | (46.05) | 80 | (21.62) | 15 | (5.56) |
|  | 5 | 75 | (23.81) | 100 | (30.77) | 80 | (21.33) | 15 | (5.00) |
| 5 | 1 | 55 | (16.67) | 95 | (24.36) | 65 | (19.40) | 0 | - |
|  | 2 | 75 | (23.44) | 120 | (36.36) | 65 | (18.84) | 20 | (8.16) |
|  | 3 | 105 | (27.63) | 125 | (34.25) | 75 | (20.27) | 20 | (7.55) |
|  | 4 | 100 | (25.00) | 140 | (35.90) | 90 | (22.50) | 20 | (6.90) |
|  | 5 | 65 | (18.06) | 130 | (34.21) | 75 | (19.48) | 0 | - |
|  | 6 | 50 | (12.99) | 140 | (35.90) | 80 | (20.78) | 35 | (12.96) |
|  | 7 | 60 | (14.81) | 170 | (42.50) | 95 | (23.46) | 30 | (9.84) |
|  | 8 | 105 | (26.92) | 145 | (37.18) | 90 | (23.08) | 10 | (3.92) |
| 6 | 1 | 95 | (30.65) | 95 | (31.67) | 70 | (23.73) | 20 | (10.26) |
|  | 4 | 50 | (15.87) | 100 | (31.75) | 70 | (22.22) | 25 | (10.20) |
|  | 5 | 120 | (40.00) | 140 | (46.67) | 75 | (24.19) | 60 | (22.64) |
|  | 6 | 135 | (43.55) | 150 | (50.00) | 70 | (23.73) | 30 | (12.50) |
|  | 7 | 45 | (14.75) | 75 | (25.42) | 65 | (22.41) | 30 | (11.76) |
| 7 | 1 | 115 | (32.86) | 75 | (21.13) | 55 | (14.86) | 20 | (7.27) |
|  | 2 | 70 | (22.58) | 60 | (19.05) | 40 | (12.50) | 15 | (5.66) |
|  | 3 | 110 | (31.88) | 75 | (21.74) | 50 | (13.89) | 20 | (7.14) |
|  | 4 | 60 | (18.18) | 70 | (20.59) | 55 | (14.67) | 25 | (9.62) |
|  | 5 | 125 | (36.23) | 80 | (23.19) | 55 | (15.94) | 15 | (5.17) |

The break-over duration is determined as the time between break-over phase onset and hoof-off for the force plate, acceleration and angular velocity algorithms. For the OMC system, the break-over duration is determined as the time between heel-off and toe-off. The stance duration is determined as the time between hoof-on and hoof-off for the force plate, acceleration and angular velocity algorithms. For the OMC system, the stance duration is determined as the time between heel-on and toe-off. Break-over duration as percentage of the corresponding stance duration is given between brackets.
