# Supplementary material for: Automatic detection of break-over phase onset in horses using hoof-mounted inertial measurement unit sensors
Source: PLoS One. 2020 May 29;15(5):e0233649. doi: 10.1371/journal.pone.0233649 (PMC7259550; doi:10.1371/journal.pone.0233649)
Supplement: S4 Table — Tables with break-over durations. Tables with break-over durations per trial in milliseconds (ms) and relative to corresponding stance duration (%) as detected with the acceleration and angular velocity algorithms, force derivative and OMC system for every hoof and gait combination. (DOCX) [file pone.0233649.s007.docx]

**Table S4: Break-over durations per trial in milliseconds (ms) and relative to stance duration (%) for right hind hoof in trot**

| Break-over duration in ms (%) for right hind hoof in trot | | | | | | | | | |
| --- | --- | --- | --- | --- | --- | --- | --- | --- | --- |
| horse ID | trial | Acceleration | | Angular Velocity | | Force Derivative | | OMC | |
| 1 | 1 | 105 | (30.43) | 140 | (40.00) | 80 | (23.19) | 25 | (9.09) |
|  | 2 | 80 | (24.24) | 130 | (36.62) | 60 | (17.65) | 20 | (7.41) |
|  | 3 | 100 | (31.25) | 135 | (40.91) | 80 | (24.24) | 25 | (9.80) |
|  | 4 | 105 | (31.34) | 135 | (39.71) | 80 | (23.53) | 30 | (10.91) |
|  | 5 | 190 | (52.05) | 165 | (42.31) | 105 | (27.63) | 30 | (10.53) |
|  | 6 | 100 | (33.33) | 155 | (50.82) | 70 | (22.95) | 40 | (19.05) |
|  | 7 | 125 | (37.88) | 125 | (36.23) | 85 | (25.00) | 50 | (17.24) |
| 2 | 1 | 70 | (25.93) | 85 | (30.91) | 55 | (18.97) | 30 | (12.00) |
|  | 2 | 55 | (22.00) | 160 | (53.33) | 45 | (15.79) | 40 | (14.81) |
|  | 3 | 45 | (17.65) | 90 | (30.51) | 40 | (13.56) | 70 | (22.58) |
|  | 4 | 25 | (10.87) | 80 | (28.57) | 60 | (21.05) | 70 | (22.95) |
|  | 7 | 35 | (14.00) | 105 | (35.00) | 60 | (20.69) | 65 | (23.64) |
| 3 | 2 | 50 | (17.86) | 85 | (30.36) | 60 | (21.05) | 25 | (11.11) |
|  | 3 | 85 | (28.81) | 85 | (29.31) | 55 | (19.30) | 25 | (11.63) |
|  | 5 | 90 | (33.96) | 80 | (29.09) | 60 | (21.43) | 20 | (8.51) |
|  | 6 | 105 | (38.89) | 90 | (31.58) | 55 | (19.64) | 20 | (8.70) |
|  | 7 | 90 | (32.73) | 85 | (30.36) | 60 | (21.05) | 20 | (8.51) |
| 4 | 1 | 85 | (27.87) | 120 | (38.71) | 65 | (19.70) | 15 | (6.00) |
|  | 2 | 85 | (28.33) | 120 | (39.34) | 55 | (18.03) | 15 | (6.25) |
|  | 3 | 75 | (25.42) | 120 | (38.71) | 60 | (19.05) | 15 | (5.77) |
|  | 4 | 30 | (10.91) | 115 | (35.94) | 65 | (20.31) | 20 | (8.16) |
|  | 5 | 80 | (26.67) | 100 | (31.75) | 60 | (18.75) | 20 | (8.16) |
| 5 | 3 | 90 | (28.13) | 85 | (26.98) | 60 | (19.67) | 0 | - |
|  | 4 | 165 | (40.74) | 240 | (59.26) | 240 | (55.17) | 0 | - |
|  | 5 | 90 | (31.03) | 95 | (30.16) | 70 | (22.22) | 35 | (15.56) |
|  | 7 | 15 | (5.00) | 95 | (28.79) | 80 | (24.24) | 70 | (35.90) |
|  | 8 | 95 | (31.15) | 60 | (22.64) | 70 | (21.54) | 30 | (13.04) |
| 6 | 2 | 90 | (32.14) | 90 | (31.58) | 65 | (21.67) | 85 | (27.87) |
|  | 3 | 20 | (7.69) | 70 | (26.42) | 45 | (16.07) | 45 | (17.65) |
|  | 4 | 40 | (18.60) | 90 | (30.51) | 55 | (19.64) | 75 | (25.42) |
|  | 5 | 30 | (11.76) | 70 | (25.45) | 50 | (18.52) | 35 | (14.89) |
|  | 6 | 270 | (66.67) | 80 | (30.19) | 55 | (20.00) | 70 | (25.93) |
|  | 8 | 185 | (51.39) | 140 | (40.58) | 55 | (17.74) | 50 | (17.24) |
| 7 | 1 | 80 | (22.22) | 70 | (23.33) | 75 | (20.55) | 55 | (19.30) |
|  | 2 | 90 | (28.13) | 60 | (21.05) | 65 | (20.00) | 30 | (11.76) |
|  | 3 | 105 | (30.88) | 60 | (21.05) | 70 | (20.59) | 35 | (11.29) |
|  | 4 | 95 | (28.36) | 65 | (22.81) | 70 | (20.90) | 50 | (18.18) |
|  | 5 | 75 | (24.19) | 65 | (22.41) | 65 | (19.40) | 20 | (7.41) |

The break-over duration is determined as the time between break-over phase onset and hoof-off for the force plate, acceleration and angular velocity algorithms. For the OMC system, the break-over duration is determined as the time between heel-off and toe-off. The stance duration is determined as the time between hoof-on and hoof-off for the force plate, acceleration and angular velocity algorithms. For the OMC system, the stance duration is determined as the time between heel-on and toe-off. Break-over duration as percentage of the corresponding stance duration is given between brackets.
