# Supplementary material for: Automatic detection of break-over phase onset in horses using hoof-mounted inertial measurement unit sensors
Source: PLoS One. 2020 May 29;15(5):e0233649. doi: 10.1371/journal.pone.0233649 (PMC7259550; doi:10.1371/journal.pone.0233649)
Supplement: Attachment — Submitted filename: Response to Reviewers B.docx [file pone.0233649.s008.docx]

**Reviewer #1:**

A useful methods manuscript. Robust methods, although in places there needs to be further clarity. Well written. Please see comments below in relation to the discussion. I feel further work here would enhance the manuscript.

1. Line 63: manual, not manually
   Line 159: Trials, Line 268: hooves
   Line 266: enrollment? Do you mean hoof-unrollment?

Thank you for reading the manuscripts with so much dedication. We have processed all your feedback in both manuscripts.

2. Line 84-85: Were these measurements from the two systems synchronized?

Yes, all three systems were hardware time synchronized which was not clearly described before. We added a short section on time synchronization in the method section and a more detailed section in the supplementary materials.

3. Line 108-111: Elevation of the heel and toe markers of 1mm may also be due to landing and breakover characteristics. e.g. the rotation of the hoof may lift the toe marker during breakover by more than 1 mm before the toe lifts.

Thank you for your feedback. We agree that 1mm is a small elevation. The toe-off and heel-off timings are determined in another study with this method (1). During the analysis, we have not seen any large differences between the force derivate and the OMC system, Table 2, nor between both algorithms and the OMC system, Table 1. Furthermore, the markers are firmly attached to the hoof which is very close to the ground and no skin artefacts are possible on this location. In addition, the horses are walked and trotted over a hard surface. Therefore, we had no reason to doubt the toe-off and heel-off timings used during this study.

4. Line 113-114: Please expand on this statement or rewrite it, as it is unclear what ‘the same preprocessing procedure’ is.
   Line 120-121: As above.

Thank you for your comment. We have added a more detailed description about the preprocessing procedure of the force plate data and IMU data in the method section. More details are also provided in the companion paper (2).

5. Line 132-133: How was a factor of 1.96 established in the first place? Was this based on something about the signal? i.e. did you quantify random noise magnitudes? Or was this done using an iterative process, based on observation of data? Please expand to provide an explanation/justification.

We used 1.96 because we assumed that the values of the IMU signal was normally distributed. The threshold is calculated to detect the upper 2.5% of the signal to be sure that no random noise was detected. We have added this description in line 153-155.

6. Table 1: There is a mistake in the way the table is displayed for walk/trot Model 5.

No this is not a mistake; it follows from the lack of significant interaction term between gait and hoof in the model. The estimated values are depicted for the two hooves and gaits separately here. We explained this in more detail in line 237-239.

7. Line 217-219: Please clearly indicate in the methods your definition of accuracy and precision (i.e. how did you measure this?).

Thank you for your noticing. We have added a definition to the methods section in line 186 and 189.

8. Line 245: The predicted values are all positive in the table?

Yes, you’re right. Thank you for noticing.

9. The overall discussion gives me, to some extent, the impression of vagueness in relation to choice of methods to compare against. It may be better at the outset to state which is the gold method for stance phase detection first.
   One might expect the most accurate stance phase detection method to be the force plate, as kinematic detection methods usually suffer from inaccurate lift off timings, thereby altering the length of the breakover phase?

Thank you for your contribution to the discussion. Indeed, it’s hard to say which method is the proper gold standard to measure the break-over onset and this is not strongly described in the literature either. There are many aspects that effects the break-over onset, for example gait, hoof/limb -conformation and horse. The force plate is considered the gold standard for kinetic measurements. In the companion paper (2), we used the force plate as gold standard for the hoof-on and hoof-off detection and we determined the stance duration as the time in between. However, determining the hoof-on and -off timings was subjective since a threshold was needed. Based on the value of this threshold, hoof-on and hoof-off timings were detected earlier or later, and stance durations became longer or shorter. The OMC system is considered the gold standard for kinematic measurements which can be used for position and rotation measurements. For the OMC system, it’s not clear whether to interpret toe-on or heel-on as hoof-on and toe-off or heel-off as hoof-off. Furthermore, to determine these toe-off and heel-off timings, a threshold of 1 mm elevation was used for both markers which is subjective but necessary. Also, we only look at the lateral side of the hoof, eliminating medial roll patterns. In this paper, we used heel-off as the break-over onset and toe-off as hoof-off moment. Since the force plate is widely used in the clinics and a flattening in the decrease of the vertical force was seen, we propose a method (first derivative) to analyse start of break-over phase from the vertical force signal. This is a novel approach to detect the break-over onset and therefore we included both force plate and OMC system as comparison. We have added this discussion point more clearly in the discussion section.

10. In order to reference your break over timings (i.e. where they occur during each stance phase) I assume you will need to know the stance phase timings? Did stance phase detection timings have an influence on break over detection methods?

Thank you for your feedback. It’s not clear to me what you mean by stance phase timings. In the companion paper (2), hoof-on and hoof-off timings were detected from the vertical force, acceleration and angular velocity signals. The stance phase durations were calculated as the time between hoof-on and hoof-off. The accuracy of break-over onset detection in relation to the stance duration was not yet described and we have added a paragraph about this in line 334-340. Please be aware that we only described break-over onset timings and not break-over durations. We expected that break-over duration changes with different walking velocities, gaits, hoof shapes and different surface properties as described in the introduction (line 46-48) and mention in the discussion section. Additionally, we now include estimated break-over durations for all methods, along with the relative duration as percentage of the corresponding stance duration, in Table S1-S4 of the supplementary materials.

11. For the hind hoof, as there is greater potential for slide and stop, did this influence your findings?

The results did not show a clear difference in performance for the front and hind hoof. With the OMC system as reference, the performance of both algorithms was better for the hind hoof. With the force derivative as reference, the performance of the acceleration algorithm was better for the front hoof in both gaits and for the angular velocity algorithm the performance was better for the front hoof in walk and for the hind hoof in trot. Furthermore, the break-over onset is measured prior to the hoof-off and after hoof-on which might the reason that no effect of slide and stop was found. In addition, valid hoof impacts were chosen by the researchers performing these measurements and steps were the horse was slipping and sliding were not included for further data collection.

12. Were the stance phase lengths all the same? Did you use just one method to identify the stance phase and then apply that to all data or did you use the timings from each individual method?

The stance durations are determined in the companion paper based on three methods; the vertical force, acceleration and angular velocity signals (2). The stance durations differ between gaits, hooves and horses since horse needed to be included as a random effect for the linear mixed model analysis. For this paper, we did not use the stance phase duration to detect the break-over onset.

13. You used a lateral marker configuration for the OMC system, how might this have been a limitation? Do all horses rotate laterally over the toe?

Studying different hoof unrollment patters was beyond the scope of this paper. We wanted to show that we could detect the onset of the break-over phase based on these signals with these algorithms. Therefore, we calculated the Euclidean norm, resulting in a one-directional signal, to conceal different hoof- and leg conformations.

14. Additionally, the accuracy and precision are reported a number of times, but no indication of the effect or magnitude of this potential ‘error’ are given. e.g. the accuracy would provide a max of a % of the stance phase difference in breakover timing either before or after the actual event etc.

Yes indeed, there are no magnitude of error are given because we only detect the break-over onset and not the duration. We have been thinking about how to calculate this magnitude of error since the break-over duration depends on hoof characteristics, gait and horse. So, an average break-over duration is not a possible here. The break-over duration can be determined for every measurement and compared with the break-over onset detection but that does not give an indication of the accuracy and precision either. Furthermore, the needed accuracy and precision depends on the gait of the horse and the application you want to use it for. Very subtle changes might be expected in case of lameness. The unrollment pattern is of more importance for hoof trimming purpose, although a more three-dimensional view would be more optimal than our presented lateral side. Furthermore, for these purposes a within horse comparison should be made and this was beyond the scope of this paper. For this paper, we only wanted to develop an algorithm that was able to detect the break-over onset.

**Reviewer #2:**

The Authors investigate an experimental set-up to automatically detect from the signals of hoof-mounted inertial measurement units (IMU) the break-over phase onset. Two algorithms are derived from the available IMU data (acceleration and angular velocity), referred to as acceleration algorithm and angular velocity algorithm. As validation of this approach, the data obtained from optical motion capture (OMC) in terms of hoof displacements and from a force plate in terms of the first time derivative of the vertical force are used. Linear mixed model analysis has been performed to assess the performance of the algorithms regarding the validation data. The manuscript is well written and structured, only minor corrections (with respect to grammar and presentation of the methodology) should be applied. Before publication, the following comments should be considered by the Authors in a revised version of the manuscript:

1) Line 22: Check word repetition ‘of a of a’ in the Abstract.
   Line 110: There is a problem with ‘to the find the...’.
   Line 117-118: The sentence should be checked (grammar).
   Line 24: Please check the manuscript for consistency regarding the use of ‘algorithm’ (singular) and ‘algorithms’ (plural). Sometimes, ‘one algorithm’ is proposed and used, sometimes ‘the algorithms’ are employed. See also Comment 17.
   Line 48 ‘..., although it can also ... indicating on...’: This sentence is hard to read in its current form. ‘although’ as well as ‘on’ in combination with ‘indicate’ have to be deleted.
   Line 61: Replace ‘helpful with determining’ by ‘helpful for determining’.

Thank you for reading the manuscripts with so much dedication. We have processed all your feedback in both manuscripts.

2) Line 33: In addition to the absolute values for accuracy and precision given in the Abstract (and in the Discussion section Lines 299-301), relative values in percent (e.g. ratio of absolute values compared to the timespan of the break-over phase) might be helpful to judge the applicability of the proposed algorithms. If the timespan of the break-over phase is not a direct outcome of the presented algorithms, at least a general estimation/indication of the typical timespan of the breakover phase should be provided.

Thank you for your suggestion. As already answered to the other reviewer, relative values for accuracy and precision cannot be calculated because the break-over duration depends on hoof characteristics, gait and horse. So, an average break-over duration is not a possible here. The break-over duration can be determined for every measurement and compared with the break-over onset detection but that does not give an indication of the accuracy and precision either. However, we have added 4 tables with break-over durations for the different hooves and gaits in the supplementary material to give the reader some feeling with break-over durations. Furthermore, in Fig. 1 a general illustration is given of the hoof movements during stance phase and the break-over onset is indicated with a dotted line.

3) Line 41: For the illustration of the explanation of the break-over phase, the Authors might already refer to Fig. 1A at this text position.

Thank you for your suggestion, we now refer to Fig. 1A in line 41.

4) Line 52: Please check if the statement ‘are able to measure’ is correct here. What are the raw data of OMC systems and IMU, i.e. rotations of the hoof or other quantities from which the rotation is derived? See also Comments 7 and 8.
   Line 53, see also Comment 6: Here, it is stated that the OMC system measures the position/displacement of markers, hence, no rotations.
   Line 54, see also Comment 6: Here, it is stated that the IMU measure acceleration and angular velocity, no rotations.

Thank you for your feedback, the OMC system measures displacement of the markers and the IMUs measure acceleration and angular velocity. So, rotations of the hoof are not literally measured, and we changed this statement to “rotations of the hoof can be derived …” (line 54-55).

5) Line 56: The sentence part with ‘artifacts and inaccuracies...’ should be replaced by e.g. ‘noise in the input data and unknown initial conditions might affect the integration and lead to inaccurate results...’

Thank you for your input, we have added your suggestion to line 60.

6) Line 93: It should be mentioned which ‘derivative’ with respect to which quantity has been used, i.e. ‘... the first derivative of this signal with respect to time’?

You’re right, we forgot to mention that the first derivative is calculated with respect to time. We have now added this description in line 113.

7) Line 96: ‘force plate and first derivative of the force plate’: Compared to the other figure caption parts, the depicted quantities, i.e. vertical force and first derivative of the vertical force, should be mentioned here in the caption, not ‘force plate and its derivative’.
   Line 97, see also Comment 12: Replace ‘OMC system’ in the figure caption by e.g. ‘rotation/displacement from the OMC system’ and add ‘(E)’. Or which quantities are depicted in Fig. 1E?

Thank you for your suggestion, we agree and changed the caption of this figure (line 115).

8) Line 130: For the equation, symbols instead of the words should be used. Subsequently, introduce/explain the symbols of the equation in the text.

We have replaced the words with symbols.

9) Line 136, see also Comment 18: Here, the two proposed algorithms, i.e. the ‘acceleration algorithm’ and the ‘angular velocity algorithm’, should be already introduced. Please state also clearly that these algorithms are compared to the validation methods, i.e. ‘OMC system data and the vertical force derivative of the force plate measurement’. In the current version, it is not clear why the new term ‘functionalities’ (better: refer to ‘algorithms’?) is used and the information in bracket is not nicely structured, i.e. mixes prediction methods with validation methods.
   Line 140: Here, the word ‘algorithms’ is used instead of the term ‘functionalities’, see also Comment 17.
   Line 161: Please consider replacing ‘functionalities’ by the algorithms, see also Comment 17.

Thank you for your comments. You’re right, the use of ‘functionalities’ does not make the methods clearer. We changed ‘functionalities’ to ‘both algorithms’ and ‘OMC and force derivative’ respectively in line 163-165 and line 200. Furthermore, we changed the sentences with the brackets to improve readability.

10) Line 141: Since the rest of the manuscript focuses on this assessment, some more details about the theory of the ‘model building and reduction procedure’ should be provided in the manuscript.

We have added a more detailed description about the linear mixed model analysis in the method section. More details are also provided in the companion paper.

11) Lines 154-156: Is this not already expressed in the paragraph before? What is the difference to Lines 145-153, where ‘reference measurement’ is used?

We apologize for the confusion, we tried to explain that first the algorithms are compared with the OMC system and force derivative over time. Thereafter, we compared the OMC system with the force derivative over time. We changed this description in line 180, we hope the description is more clearly now.

12) Line 187: Explain the term ‘models’ in more detail.

We meant to say that the residuals of the linear mixed models were normally distributed. We hope that the description is now easier to understand (line 233).

13) Table 1: What are Models 1 to 3, why starting with Model 4? If the Authors change the model names (numbers), please adapt also references to these models in the rest of the manuscript.

We forgot to change the numbering after rewriting the manuscripts. Thank you for noticing.

14) Although a Conclusion section is optional for this journal, the Authors should think about splitting the Discussion section into ‘Discussion’ and ‘Conclusion’, e.g. starting from Line 295.

We agree, we have added a conclusion section to both manuscripts.

15) Fig. 1: Place the legend ‘first derivative’ also in the area of Fig. 1D so that this legend is not associated with (the area of) Fig. 1E.

Thank you for your suggestion, we have changed the location of the text in the figure.

**References:**

1.	Braganca FM, Bosch S, Voskamp JP, Marin-Perianu M, Van der Zwaag BJ, Vernooij JCM, et al. Validation of distal limb mounted inertial measurement unit sensors for stride detection in Warmblood horses at walk and trot. Equine Vet J. 2017;49(4):545-51.

2.	Tijssen M. A method for automatic hoof-event detection in horses based on hoof-mounted inertial measurement units. Submitted to PLOS ONE. 2020a, companion paper.
